# MIDnight for the Monetary Incentive Delay Task as an Endophenotype?

**DOI:** 10.1016/j.bpsgos.2026.100729

**Published:** 2026-05-11

**Authors:** Mitul A. Mehta, Peter C.T. Hawkins

**Affiliations:** Department of Neuroimaging, Institute of Psychiatry, Psychology and Neuroscience, King’s College London, London, United Kingdom

The ability to robustly index reward-related brain activity with a simple functional magnetic resonance imaging (fMRI) task—the monetary incentive delay (MID) task—had a major impact when introduced more than 25 years ago (1). The main subcortical activations (the initial study described monetary reward–related activation to be in the ventral striatum, matching studies in experimental animals using primary rewards) have been reproduced many times, and the activations are sensitive to multiple disorders, across development, and to various interventions (2,3). This has made the task a very attractive candidate for use as a marker of brain function during reward processing. Indeed, objective neural markers that index the biological processes that give rise to different psychiatric symptoms are important for the field as they offer the potential to identify pathological subgroups that cut across current categorical boundaries, the brain circuits that underlie different symptom profiles, and crucially identify treatment targets for those symptoms. Approaching treatment development in this way may prove more beneficial than attempting to address psychiatric diagnostic labels, which rely on assigning observed mental health presentations to a symptom-based classification that is, in practice, heterogeneous, diagnostically overlapping, and often poorly related to available measures of neurobiology.

Barendse *et al.* (4), in a recently published article in *Biological Psychiatry: Global Open Science*, examined anticipation of reward and loss in the MID task in a group of patients with psychotic and bipolar disorders, their first-degree relatives, and control participants to test whether MID ventral striatal activation meets the criteria of an endophenotype. The endophenotype concept in psychiatry, as described by Barendse *et al.* (4), is a means of identifying measurable biological markers that are more reflective of the underlying genetic architecture than the clinical diagnoses attributed to observed symptoms. They proposed 5 criteria: the endophenotype must be heritable, associated with illness in the population, state independent, co-segregating with illness within families, and present in unaffected relatives at higher rates than in the general population.

The authors scanned 97 participants with bipolar disorder, 29 participants with various psychosis presentations, 51 relatives, and 66 control participants. They also calculated polygenic risk scores for bipolar disorder, schizophrenia, and depression. The results were a resounding negative. The groups did not differ in the degree of ventral striatal activation for the reward anticipation versus the no-reward anticipation contrast. For the same contrast, there was no relationship with depressive, manic, or positive or negative schizophrenia symptoms. Thus, 4 of the 5 endophenotype criteria were not met. While polygenic associations are not a test of heritability, they can establish genetic associations. Here, the results were negative for all 3 polygenic risk scores again.

These null findings are not isolated within the wider MID literature. As noted by Barendse *et al.* (4), a recent meta-analysis of the MID task in participants with bipolar disorder did not evidence differences from control participants in ventral striatal activation (5). This aligns with the findings from Barendse *et al.* (4) in their largest group of patients (*n* = 97). Meta-analyses of ventral striatum activation in schizophrenia do suggest consistent hypoactivation in the ventral striatum during reward anticipation, leaving open the possibility that Barendse *et al.* (4) were simply underpowered to detect such a difference (*n* = 29), especially as part of the larger patient group. The lack of correlation with symptoms in this study runs counter to some earlier studies that found relationships with both positive (3) and negative/anhedonic (6) symptoms. However, larger, more recent studies have failed to replicate this relationship in schizophrenia (7), and the lack of difference in activation between the 51 first-degree relatives and control participants is still in keeping with the overall conclusion that the endophenotype proposal is not supported.

We must then question whether the MID ventral striatal activation is an informative measure for use in clinical populations. The typical outcomes of the MID task are neural responses to reward anticipation and receipt, but there may be other candidate outcomes. For example, the task does not directly assess effort allocation or reward learning, which are critical aspects of motivated behavior and are impaired in psychotic and bipolar disorders (8). Regardless of which outcome measure is used, we contend that there remains a role for assessment of reward processing in treatment assessment and prediction. While the MID may not meet the high bar for an endophenotype, the use of functional biomarkers may have potential for stratifying patients with reward processing differences and evidencing functional target engagement of interventions.

While such markers do not require heritability or co-segregation, they still need to be reliable and linked to clinically meaningful outcomes. These are not insignificant challenges. It is now well established that commonly used fMRI tasks show only moderate reliability. Poor reliability will constrain the utility to detect stable relationships with other outcomes, such as symptom ratings.

Potential routes to improved sensitivity and reliability are being developed. First, Sacchet *et al.* (9) have used a machine learning approach to identify features of individualized topographies of subcortical activations in the MID task to predict depression and anxiety symptoms in patients with anhedonia. A similar approach is yet to be tested in psychotic or bipolar disorders, and limited attempts have been made to incorporate ecologically valid paradigms. Second, our own unpublished work has indicated that there are at least 2 different response styles in the MID task, captured by differential patterns of reward anticipation and receipt activity when analyzed together (10). By capturing such heterogeneity, there is potential for improved sensitivity to diagnosis, symptoms, and interventions. Despite these advances, there may be limits to the degree to which symptoms and their cognitive, social, and environmental influences may be reduced to responses to monetary or other rewarding stimuli in a scanner.

The assessments of ventral striatum activation during the MID task in Barendse *et al.* (4) may spell the end of its use as an endophenotype in the mood-psychosis spectrum, but it has not given up all its secrets yet.

## References

[1] Knutson B., Westdorp A., Kaiser E., Hommer D. (2000). FMRI visualization of brain activity during a monetary incentive delay task. NeuroImage.

[2] Schwarz K., Moessnang C., Schweiger J.I., Baumeister S., Plichta M.M., Brandeis D. (2020). Transdiagnostic prediction of affective, cognitive, and social function through brain reward anticipation in schizophrenia, bipolar disorder, major depression, and autism spectrum diagnoses. Schizophr Bull.

[3] Nielsen M.Ø., Rostrup E., Wulff S., Bak N., Lublin H., Kapur S., Glenthøj B. (2012). Alterations of the brain reward system in antipsychotic naive schizophrenia patients. Biol Psychiatry.

[4] Barendse M., Bilder R., Snijders A. (2026). Reward-related brain function as an endophenotype in the mood-psychosis spectrum. Biol Psychiatry Glob Open Sci.

[5] Long X., Wang X., Tian F., Cao Y., Xie H., Jia Z. (2022). Altered brain activation during reward anticipation in bipolar disorder. Transl Psychiatry.

[6] Radua J., Schmidt A., Borgwardt S., Heinz A., Schlagenhauf F., McGuire P., Fusar-Poli P. (2015). Ventral striatal activation during reward processing in psychosis: A neurofunctional meta-analysis. JAMA Psychiatry.

[7] Carruzzo F., Kaliuzhna M., Kuenzi N., Geffen T., Katthagen T., Schlagenhauf F., Kaiser S. (2024). Striatal response to reward anticipation as a biomarker for schizophrenia and negative symptoms: Effects, test–retest reliability, and stability across sites. Schizophr Bull.

[8] Culbreth A.J., Moran E.K., Kandala S., Westbrook A., Barch D.M. (2020). Effort, avolition and motivational experience in schizophrenia: Analysis of behavioral and neuroimaging data with relationships to daily motivational experience. Clin Psychol Sci.

[9] Sacchet M.D., Valenti J.L., Keshava P., Walsh S.W., Smoski M.J., Krystal A.D., Pizzagalli D.A. (2025). Individualized functional brain mapping machine learning prediction of symptom-change resulting from selective kappa-opioid antagonism in an anhedonic sample from a Fast-Fail trial. J Mood Anxiety Disord.

[10] Sambuco A., Lupo A., Passiatore R., Hawkins P., Selvaggi P., Antonucci L. (2025). Individual reward styles predict brain and behavioral responses to dopaminergic challenges. AIP Sperimentale 2025, 31° Congresso annuale. https://indico.sissa.it/event/159/book-of-abstracts.pdf.

